# Foreign Subtitles Help but Native-Language Subtitles Harm Foreign Speech Perception

**DOI:** 10.1371/journal.pone.0007785

**Published:** 2009-11-11

**Authors:** Holger Mitterer, James M. McQueen

**Affiliations:** 1 Max Planck Institute for Psycholinguistics, Nijmegen, The Netherlands; 2 Behavioural Science Institute and Donders Institute for Brain, Cognition & Behaviour, Centre for Cognition, Radboud University Nijmegen, Nijmegen, The Netherlands; New York University, United States of America

## Abstract

Understanding foreign speech is difficult, in part because of unusual mappings between sounds and words. It is known that listeners in their native language can use lexical knowledge (about how words ought to sound) to learn how to interpret unusual speech-sounds. We therefore investigated whether subtitles, which provide lexical information, support perceptual learning about foreign speech. Dutch participants, unfamiliar with Scottish and Australian regional accents of English, watched Scottish or Australian English videos with Dutch, English or no subtitles, and then repeated audio fragments of both accents. Repetition of novel fragments was worse after Dutch-subtitle exposure but better after English-subtitle exposure. Native-language subtitles appear to create lexical interference, but foreign-language subtitles assist speech learning by indicating which words (and hence sounds) are being spoken.

## Introduction

Listeners have difficulty understanding unfamiliar regional accents of their native language [1]. This is in part because the speech sounds of the accent mismatch those of the language standard (and/or with the listener's own accent). Listening difficulty is magnified when the unfamiliar regional accent is in a foreign language: The unusual foreign vowels and consonants may mismatch more with native sound categories, and may even fail to match any native category [2]. This situation arises, for example, when we watch a film in a second language. Imagine a American listener, fluent in Mexican Spanish, watching El Laberinto del fauno [Pan's Labyrinth, 3]. She may have considerable difficulty understanding the European Spanish if she is unfamiliar with that language variety. How might she be able to cope better? We argue here that subtitles can help. Critically, the subtitles should be in Spanish, not English. This is because subtitles in the language of the film indicate which words are being spoken, and so can boost speech learning about foreign speech sounds.

Perceptual learning studies show that speech processing in the listener's native language can be retuned by lexical knowledge. Specifically, listeners can learn to interpret an ambiguous phoneme on the basis of disambiguating lexical contexts [4]–[6]. In a typical experiment, an ambiguous segment midway between/s/and/f/(“?”) might appear either in sequences such as *hor?* or, for another group of listeners, in sequences such as *gira?*. Since *horse* and *giraffe* are words and *horf* and *giras* are not, the first group learns to perceive the ambiguous sound as/s/, and the second group learns that it is/f/. Lexically-biased exposure thus results in shifts in the perceptual/s/-/f/category boundary. What is learned during these exposure conditions is used to interpret previously unheard words [7]. That is, listeners who have heard sequences such as *hor?* would subsequently tend to interpret *li?* as *lice* rather than *life*, while those who have heard *gira?* would tend to recognize *li?* as *life*. Lexically-guided retuning of speech-sound categories therefore benefits comprehension. Lexical knowledge helps listeners adapt to the exposure talker's unusual speech, and thus allows them to understand that talker better.

The present study addressed whether this kind of perceptual learning comes to the aid of a listener confronted with an unfamiliar foreign regional accent. Subtitles indicate which words are being spoken, and so could boost lexically-guided learning about foreign speech sounds. Given the prior work on lexical retuning within the native language [4]–[8], and the suggestion that adaptation to foreign-accented speech is in part lexically driven [9], there may be lexical retuning also in second-language listening. That is, listeners may be able to retune speech-sound categories based on their knowledge about how foreign words ought to sound (imagine, e.g., a Spanish listener fluent in English learning about “?” in English *hor?/gira?* experiments). We tested that prediction here, but in a novel way, by asking whether subtitles can support learning about unfamiliar regional accents in a foreign language. If so, this would suggest not only that subtitles can help foreign speech understanding, but also that one way in which they do so is through the mechanism of lexically-guided perceptual learning.

Facilitation of adaptation to foreign regional accents by subtitles would show for the first time that lexically-guided retuning applies in second-language perception. It would also constitute evidence that lexically-guided retuning scales up to the task of adapting to real-speech variation. Previous studies have used artificially-created ambiguous phonemes [4]–[8], [10], or synthetic or artificially-distorted speech [11], [12]. In addition, subtitle-driven adaptation would suggest that retuning of phonemic categories can be induced by orthographic information, just as retuning can be induced by concurrent visual speech [e.g.], [from lipreading, 6,13]. Unlike visual speech, however, subtitles provide only abstract representations of speech sounds and lexical forms. Can abstract orthographic information nevertheless influence learning in speech perception?

Dutch participants were asked to watch videos containing unfamiliar regionally-accented English, with or without subtitles. Critically, both English and Dutch subtitles were used. This made it possible to compare the effects of subtitles in the language spoken in the videos with the effects of subtitles in the observers' native language. Previous research on subtitles focused on the effects of native and foreign subtitles on global measures of plot memory, grammar and vocabulary learning, or viewers' attitudes. The results suggested that foreign subtitles are helpful but that native-language subtitles provide no benefit or less benefit [14]–[18]. Only one study focused on phonological processing; negligible benefits for non-native subtitles were found [19]. Clear predictions can nevertheless still be made about the effects of subtitle language on speech learning. If lexically-guided retuning operates in second-language listening, and is open to any influence from subtitles, then the influence should depend on the language of the subtitles. English subtitles should give our observers most of the words in the speech stream (not all, because subtitles are seldom literal transcriptions). These printed English words can provide an additional source of information about the words being spoken, and hence about the sounds being heard, and so ought to reinforce lexically-guided learning. In contrast, native Dutch subtitles may be easier for the observers to read, but provide misleading information about the phonological forms being spoken. So Dutch subtitles should interfere with perceptual learning.

We thus tested whether subtitles help or hinder adaptation to an unfamiliar regional accent in a second language. Dutch participants, fluent in English, watched 25 minutes of video material with either strongly-accented Australian English [an episode of the Australian sitcom Kath & Kim, 20] or strongly-accented Scottish English [excerpts from the British movie Trainspotting, 21]. In each case, separate groups had either English, Dutch, or no subtitles. After this exposure, all six groups were asked to repeat back excerpts from both the Australian and the Scottish material. The groups exposed to Scottish English thus provide no-exposure control data for the Australian English excerpts, and vice versa. Because the focus was on adaptation in listening, the excerpts were audio only. There were 160 excerpts in total. Eighty excerpts (spoken by the main characters in each video) were taken from the exposure material (forty from each source). Eighty excerpts were completely new, but from the same speakers (again, forty Scottish and forty Australian excerpts). The latter material in particular allowed us to assess how well listeners adapted to the accent during exposure.

## Results

We present analyses of the proportion of words repeated correctly overall (see Materials and Methods). We scored how many words (content and function words) were repeated correctly per excerpt. Table 1 shows the proportion of correctly repeated words per excerpt, split by old and new items and by accent type. We predicted success on individual trials using a linear-mixed effect model [22] with a logit as a linking function because of the limited range of the dependent variable ([0, 1]). Individual data points were predicted with crossed fixed and random effects. For categorical predictor variables, one level is mapped onto the intercept and binary dummy variables are created for the other levels. To best estimate the effect of subtitling, we mapped the no-subtitles condition onto the intercept.

**Table 1:** Mean proportions of correctly repeated words and percentage gain over the control condition.

	Type of English			
	Australian English		Scottish English	
Type of Subtitle	New Items	Old Items	New Items	Old Items
No Subtitles	0.77 (+6%)	0.79 (+8%)	0.84 (+4%)	0.82 (+6%)
English Subtitles	0.79 (+9%)	0.85 (+14%)	0.86 (+6%)	0.84 (+8%)
Dutch Subtitles	0.72 (+1%)	0.79 (+8%)	0.83 (+3%)	0.84 (+8%)
Control	0.71	0.71	0.80	0.76

Although the Australian English proved overall more difficult to repeat than the Scottish English (in the control conditions, 71% of the Australian and 78% of the Scottish words were repeated correctly), accent type did not modulate any other effects. Neither the interaction of Exposure Materials with Subtitles Condition and Old/New (p_min_>0.2) nor the interaction of Exposure Materials with Subtitles Condition (p_min_>0.3) produced significant regression weights. The raw values in Table 1 may appear to suggest that performance was especially bad when participants who had been exposed to Dutch subtitles with the Australian material had to repeat new materials. The comparisons to the control conditions, however, show that the pattern of learning effects is similar for both material sets, if somewhat more pronounced for the Australian materials.

**Figure 1:**
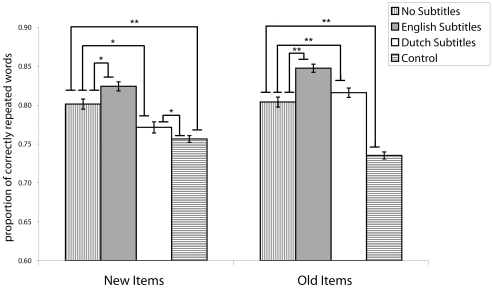
Mean proportions of correctly repeated words by Dutch listeners. The data are collapsed over exposure/test regional accent (Scottish or Australian English), for new and previously heard items and for each of the exposure conditions: no subtitles, English subtitles, or Dutch subtitles, or no prior exposure to the test accent (Control). Error bars are ±1 Standard Error of the mean. ** = p<.01; * = p<.05. There were three key findings: (1) Exposure to an unfamiliar foreign regional accent improves speech understanding; (2) Native-language subtitles help recognition of previously heard words but harm recognition of new words; (3) Foreign-language subtitles improve repetition of previously heard and new words, the latter demonstrating lexically-guided retuning of perception.

We therefore collapsed over exposure materials (see Figure 1) and analyzed the proportion of correctly repeated words with condition (English subtitles, Dutch subtitles, No subtitles, Control) and repetition (Old vs. New items) as factors. The effects of the subtitles were different for old and new items (*p*<0.01). On the old items, exposure to speech alone (in the No-subtitles condition) yielded better performance than in the Control condition (*β*_control_ = −0.44, *p*<0.01, *β*s indicate differences from the No-subtitles condition). Furthermore, more words were repeated correctly after English- and Dutch-subtitle exposure than after No-subtitle exposure (*β*_EnglishSubtitles_ = 0.34, *p*<0.01, *β*_DutchSubtitles_ = 0.24, *p*<0.01). For the new items, the pattern was different. More words were repeated correctly in the English-subtitles condition than in the No-subtitles condition (*β*_EnglishSubtitles_ = 0.19, *p*<0.05). Furthermore, performance in the No-subtitles condition was better than in the Control condition (*β*_control_ = −0.33 *p*<0.01). But repetition performance in the Dutch-subtitles condition was worse than in the No-subtitles condition (*β*_DutchSubtitles_ = −0.17, *p*<0.05). Nevertheless, Dutch-subtitle exposure led to more words being repeated correctly than in the Control condition (*β*_DutchSubtitles vs control_ = 0.16, *p*<0.05). The latter effect may seem surprising, given the overall difference of only 1.5% between the two groups. An analysis of the distributions of responses in those conditions revealed, however, that the difference between the two groups lies mainly in the proportion of completely correct repetitions, which were far more likely in the Dutch subtitle group. Given this distribution, the non-linear logistic transformation [validly, cf. 23] increases the difference between these two groups in logistic space, producing a significant difference.

Finally, we tested the source of the effects on new items. Although these items had not been presented during the exposure phase, 69% of the words in these novel phrases had been spoken in the exposure material. Participants who had heard and seen these words before could thus have performed better than other participants because of word-specific learning. To test the possible role of word-specific learning, we calculated the item-specific benefit of the No-subtitles group over the control group as well as the benefit of the English-subtitles group over the No-subtitles group. These measures were then correlated with the proportion of words in each new item that also were in the exposure materials. The proportion of words present during exposure influenced the item-specific benefit of the no-subtitles group over the control group (*r*(78) = 0.26, *p*<0.05), but not the benefit of the English-subtitles group over the no-subtitles group (*r*(78) = 0.02, *p*>0.5). The correlational analysis thus indicated that word-specific learning may influence the overall adaptation effect—the difference between the Control and No-subtitle conditions—, but not the additional benefit for the English subtitles condition. The benefit due to the English subtitles thus appears to reflect generalization of learning across the lexicon.

## Discussion

We asked two questions. First, we tested whether audiovisual exposure allows listeners to adapt to an unfamiliar foreign accent. Second, we asked whether subtitles can influence this process. Our results show that this kind of adaptation is possible, and that subtitles which match the foreign spoken language help adaptation while subtitles in the listener's native language hinder adaptation.

The differences between the experimental and the control conditions speak to our first question. They show that listeners can adapt to an unfamiliar regional accent in a second language after only brief audiovisual exposure. Note that listeners in the control condition for each accent were exposed to the other accent. To assess whether differences relative to the control condition may be due to inhibition caused by hearing a different accent at test than during exposure, we estimated the upper bound of this inhibition from the data on the new items. The listeners in the Dutch-subtitles condition heard the same accent at exposure and test and yet they performed only slightly—but significantly—better (1.5%) than the listeners in the control condition. This small difference shows that accent mismatch between exposure and test in the control condition resulted in very little inhibition, if indeed any at all. All larger differences between experimental and control conditions must be due instead to facilitatory adaptation to the exposed accent. This adaptation is likely to reflect not only adjustments to the unfamiliar speech which are based on phonological learning driven by lexical knowledge (lexically-guided retuning [4]) but also adjustments which are based on other information sources. Our correlational analysis showed that word-specific learning seems to contribute to this effect. Additionally, sub-lexical phonological knowledge, concurrent visual information (i.e., lip-read speech; [13]), and acoustic-phonetic information in the speech signal itself [24], [25] have also been shown to enable perceptual learning in speech and are likely to contribute here as well. It is hence likely that both “supervised” (e.g., lexically-driven) and “unsupervised” (e.g., signal-driven) learning mechanisms are involved. Future research will be required to establish the relative importance of these mechanisms. The current study nevertheless establishes that rapid adaptation to accented foreign speech is at least possible. That is, this kind of learning is not limited to recognition of speech with a foreign accent in the listener's native language [as shown by 9], [26]; it is also possible with regionally-accented speech in a foreign language.

Our second question—about the roles of subtitles in adaptation—was our main focus. It was addressed by the comparisons between the different subtitle exposure conditions. Perceptual adaptation was enhanced by subtitles that were in the same language as the accented speech. Adaptation effects, and their enhancement by English subtitles, were found for old and new items. These adaptation and enhancement effects on the old items suggest that listeners were better able to recognize the foreign-accented speech during exposure and to encode those phrases in memory. The effects on the new items, however, are the key results. The adaptation effect shows that listeners were able to retune their perceptual categories to characteristics of the exposure speakers, leading to long-term changes in speech perception. The enhancement of this adaptation by English subtitles suggests in turn that the retuning benefited from listeners knowing what words they were hearing. This indicates that the listeners were using lexical knowledge to retune phonetic perception. Although it is possible that some of the adaptation and enhancement effects reflect word-specific learning (many of the words in the new items had been heard and seen before, in other exposure phrases), the lack of an effect of exposure-test repetition in the correlational analyses is inconsistent with the hypothesis that the effect of the English subtitles is due to word-specific learning. Instead, it appears that the enhancement caused by the English subtitles reflects, at least in part, retuning of prelexical perceptual categories [4]. Retuning at the prelexical level benefits the recognition both of words that have been heard before and of completely novel words containing the retuned sounds [7].

The most dramatic aspect of our results was how different the effects of the English and Dutch subtitles were. English subtitles were associated with the best performance on both old and new items. But although Dutch subtitles also enhanced performance on the old items, they led to worse performance on the new materials. The participants apparently used the semantic information in the Dutch subtitles when listening to the English [cf. 18], and did not ignore the English speech. Indeed, the Dutch subtitles appear to have helped the participants to decipher which English words had been uttered, as seen in the benefit on recognition of previously heard materials. But this did not allow participants to retune their phonetic categories so as to improve their understanding of new utterances from the same speaker. Why was this the case? Phonological knowledge is automatically retrieved during print exposure [27], so the Dutch subtitles provided phonological information that was inconsistent with the spoken English word forms. This would weaken the influence of English lexical-phonological knowledge on perceptual learning. The account based on the mechanism of lexically-guided retuning thus explains both the positive effect of subtitles in the language of the film and the negative effect of subtitles in the perceiver's native language. According to this account, the orthographic information in subtitles can influence learning in speech perception either in a facilitatory manner (as when the English subtitles indicated which words, and hence phonemes, were being spoken) or in an inhibitory manner (as when the Dutch subtitles specified the wrong phonological information).

Two points follow from the conclusion that the subtitle effects reflect lexically-guided retuning of perceptual categories. First, it would appear that lexically-guided learning can occur with real speech in a naturalistic setting. The phenomenon seems not to be restricted to the psycholinguistic laboratory. Second, this conclusion is consistent with the claim that lexically-guided retuning contributes to the way native listeners adapt to foreign-accented speech [9]. Importantly, however, the present findings show for the first time that this kind of perceptual learning is not restricted to native listening: It also occurs in second-language listening.

Our demonstration of perceptual learning about speech sounds in a second language has implications for both theory and practice in second-language acquisition. It has been suggested that certain aspects of language acquisition are fundamentally different in a second as opposed to a first language [28], [29]. With respect to speech recognition, however, the same perceptual-learning mechanism appears to apply in first- and second-language processing. This is in a way not surprising as this type of learning does not seem to be domain specific; similar learning effects have been observed with letters [30] and with color categories [31]. Given that prior knowledge appears to modulate perception across cognitive domains, such as speech perception, reading, and color perception, it is not surprising that this form of learning applies in both first- and second-language acquisition. Nevertheless, our data rule out any account in which learning a second language draws on resources which do not overlap with those used in learning a first language.

As we used real subtitles, our results also have practical implications. Although the use of real subtitles meant that the listeners did not get a word-by-word transcription of the dialogue, it allows us to generalize our results to visual media exposure outside the laboratory. It appears that the largest benefit from this kind of real-world exposure, in the recognition of regional accents in a second language, comes from the use of subtitles in that language. But foreign-language subtitles are not what television viewers and filmgoers are familiar with. In many European countries (e.g., Germany) there is considerable public concern about international comparisons of scholarly achievements [e.g., 32]. Yet viewers are denied access to foreign-language speech, even on publicly-financed television programs. Instead, foreign languages are dubbed. In countries which use subtitles instead of dubbing (e.g., the Netherlands), only native-language subtitles are available, so again listeners are denied potential benefits in speech learning. Native-language subtitles are obviously essential for listeners who do not already speak a second language, and may thus be the only practical solution in cinemas. With the advent of digital television broadcasting, however, it is now possible to broadcast multiple audio channels and multiple types of subtitles. We suggest that it is now time to exploit these possibilities. Individuals can already take matters into their own hands, however. It is often possible to select foreign subtitles on commercial DVDs. So if, for example, an American speaker of Mexican Spanish wants to improve her understanding of European Spanish, we suggest that she should watch some DVDs of European Spanish films with Spanish subtitles.

Such same-language subtitles indicate which words are being spoken, and so, via the lexically-guided retuning mechanism, they can boost speech learning and hence facilitate language understanding. This happens even though the words are in a foreign language and the speech is in an unfamiliar foreign regional accent. It might seem remarkable that lexically-guided learning can operate under these non-ideal conditions, yet it is under these sorts of circumstances that speech learning can yield the greatest benefits in speech understanding.

## Materials and Methods

121 participants from the subject pool of the Max Planck Institute for Psycholinguistics participated in the experiment. This research was carried out in accordance with Dutch law and adhered to the guidelines of the American Psychological Society. The research was exempt under Dutch legislation for ethical review. Participants gave written informed consent to their participation after they were completely informed about the nature of the study, specifically that they could be exposed to television excerpts with potentially offensive language. One participant declined to participate after being so informed.

The participants were native speakers of Dutch studying at the Radboud University Nijmegen, with good command of spoken and written English [e.g., English reading materials are used in Dutch university curricula, and participants from this pool have, on average, 7–8 years of English education, see 33]. They had not been to Scotland or Australia for longer than two weeks, so were unfamiliar with Scottish and Australian English. Six groups of 20 participants watched either the Australian or the Scottish material, each presented with English, Dutch or no subtitles.

Participants watched either a 25 min episode of *Kath* & *Kim* (Season 1, Episode 5) or a shortened 25 min version of *Trainspotting*. *Kath* & *Kim* is an Australian sitcom about a mother (Kath) and a daughter (Kim) living in suburban Melbourne. *Trainspotting* is a movie about a Scottish drug addict named Renton and his group of friends; in the shortened version, the potentially most disturbing episodes were edited out. For *Trainspotting*, we used the English and Dutch subtitles available on the DVD. For *Kath* & *Kim*, only English subtitles were available. Dutch subtitles were created by translating the English subtitles into Dutch and presenting them with the same timing and display format as the English subtitles.

After this exposure, participants were asked to repeat back 80 audio excerpts from each source, spoken by the main characters (Kath from *Kath* & *Kim*; Renton from *Trainspotting*). Excerpts were phrases from the movies that were bounded by pauses. Half came from the exposure material (old items), and half were completely new items, taken either from unused parts of *Trainspotting* or from another *Kath* & *Kim* episode (Season 1, Episode 2). Every participant had to repeat back all 160 utterances, so that all participants exposed to Australian (i.e., collapsed across subtitle conditions) acted as a no-exposure control for the Scottish participants, and vice versa. Accent was blocked, such that participants heard either the Scottish and then the Australian excerpts, or the reverse. Old and new items were randomly mixed within these blocks.

On each trial, participants heard a warning tone 750 ms before stimulus onset. The excerpt was presented twice with a stimulus onset asynchrony of 3.5 times its duration. Participants then had 7.5 times the excerpt's duration to react before the next trial started. They were instructed to respond to the first presentation of the excerpt as fast as possible, but only if they were certain about what they heard. After the second presentation, they were encouraged to repeat back any words they might have heard. It was stressed that there was no need for them to imitate the accent of the speaker. Participants' responses were recorded using a microphone and stored on DAT. These were scored for repetition accuracy offline by two judges who were naive as to the purpose of the experiment.
